# Exploiting Polynomial Chaos Expansion for Rapid Assessment of the Impact of Tissue Property Uncertainties in Low‐Intensity Focused Ultrasound Stimulation

**DOI:** 10.1002/bem.70004

**Published:** 2025-03-12

**Authors:** Kemal Sumser, Rob Mestrom, Yunus Emre Tuysuz, Margarethus Marius Paulides

**Affiliations:** ^1^ Care & Cure Lab of the Electromagnetics Group (EM4Care+Cure), Department of Electrical Engineering Eindhoven University of Technology Eindhoven The Netherlands; ^2^ Department of Electrical and Electronics Engineering Middle East Technical University Ankara Turkey; ^3^ Department of Radiotherapy Erasmus University Medical Center Cancer Institute Rotterdam The Netherlands

**Keywords:** focused ultrasound, Monte Carlo method, neuromodulation, polynomial chaos expansion, ultrasound

## Abstract

Neuromodulation with low‐intensity focused ultrasound (LIFUS) holds significant promise for noninvasive treatment of neurological disorders, but its success relies heavily on accurately targeting specific brain regions. Computational model predictions can be used to optimize LIFUS, but uncertain acoustic tissue properties can affect prediction accuracy. The Monte Carlo method is often used to quantify the impact of uncertainties, but many iterations are generally needed for accurate estimates. We studied a surrogate model based on polynomial chaos expansion (PCE) to quantify the uncertainty in the LIFUS acoustic intensity field caused by tissue acoustic property uncertainties. The PCE approach was benchmarked against Monte Carlo method for LIFUS in three different head models. We also investigated the effect of the number of PCE samples on the accuracy of the surrogate model. Our results show that the PCE surrogate model requires only 20 simulation samples to estimate the mean and standard deviation of the acoustic intensity field with high accuracy compared to 100 samples needed for Monte Carlo method. The root mean squared percentage error (RMSPE) in the mean acoustic intensity field was less than 1.5%, with a maximum error of less than 0.5 W/cm^2^ (< 1% of the focus peak intensity in water), while the RMSPE in the standard deviation was less than 9%, with a maximum error of less than 0.3 W/cm^2^. The accuracy of the PCE surrogate model, and the limited number of iterations it requires makes it a promising tool for quantifying the uncertainty in the acoustic intensity field in LIFUS applications.

## Summary

- Uncertainties in acoustic properties impact computational model predictions in neuromodulation using low‐intensity focused ultrasound.
- Uncertainties in the predicted acoustic intensity field can be accurately and rapidly quantified by creating a surrogate model based on Polynomial Chaos Expansion (PCE)
- This framework can be extended to evaluate multiple parameter uncertainties in acoustic modeling, as well as thermal property uncertainties for thermal safety assessment.

## 1 Introduction

Neuromodulation with low‐intensity focused ultrasound (LIFUS) is an emerging field that holds great promise for the noninvasive treatment of neurological disorders (Fomenko et al. 2018). However, the success of this technique heavily relies on how accurately specific brain regions can be targeted with a sound pressure field. To assess targeting from a computational modeling point of view, precise knowledge of the acoustic properties of the tissue being treated is required. While magnetic resonance imaging (MRI) is a highly accurate method for segmenting tissue, determining acoustic properties and monitoring treatments, it is also an expensive modality that is not feasible for routine clinical use (Olinger et al. 2023; Fomenko and Lozano 2019; Conti et al. 2020). By leveraging the power of computational modeling, we can investigate the effect of tissue acoustic property uncertainties on the predicted acoustic intensity field. Better knowledge of the predicted acoustic intensity field and how this relates to the aforementioned uncertainties will benefit the targeting of neuromodulation, which, in turn, will help to better relate to the clinical outcome of focused ultrasound.

Monte Carlo simulations systematically investigate the effect of various uncertainties by randomly varying these uncertainties within their expected ranges during each virtual experiment (Metropolis and Ulam 1949). For example, in the context of neuromodulation with low‐intensity focused ultrasound, the tissue acoustic properties, such as density and speed of sound, can vary from patient to patient and within a single patient's brain (Aubry et al. 2003). By modeling these variations as random perturbations, the Monte Carlo method can simulate the possible range of acoustic property values and their impact on the predicted acoustic intensity field during ultrasound treatment. Running hundreds of such simulations allows for generating a distribution of possible outcomes, which provides an estimate of the uncertainty in the predicted acoustic intensity field. This analysis can be useful in identifying the impact of acoustic property uncertainties on stimulation accuracy and explaining the differences seen in different studies or subjects. The main drawback of the Monte Carlo method is that it can be computationally expensive for systems with a large number of uncertain variables. For the case of acoustic modeling for LIFUS, a single acoustic simulation can take several seconds to several minutes, depending on the model size, and the application frequency. Analyzing a certain effect usually requires at least hundreds of simulations, which take at least several hours. Furthermore, if a different effect needs to be investigated, the computations have to be repeated. Hence, an alternative method to overcome these shortcomings is desired.

Polynomial chaos expansion (PCE) is a method for representing a random variable in terms of a polynomial function of other random variables (Crestaux et al. 2009). PCE can be used to propagate uncertainty through complex models, and to generate surrogate models that are more efficient to evaluate than the original models. Successful applications of PCE to evaluate the effect of uncertainties have been shown in a variety of fields (Witteveen et al. 2007; Perkó et al. 2014; Groen et al. 2023).

In this study, we studied surrogate modeling using Polynomial Chaos Expansion (PCE) to rapidly analyze how the multiple uncertainties in tissue acoustic properties translate into an uncertainty in the predicted acoustic intensity field. We chose the PCE method to model multiple uncertainties. We have compared and evaluated our method against the traditional Monte Carlo method using three different head models. We have also investigated the effect of number of samples on the accuracy of the surrogate modeling.

## 2 Methods

**Figure 1:**
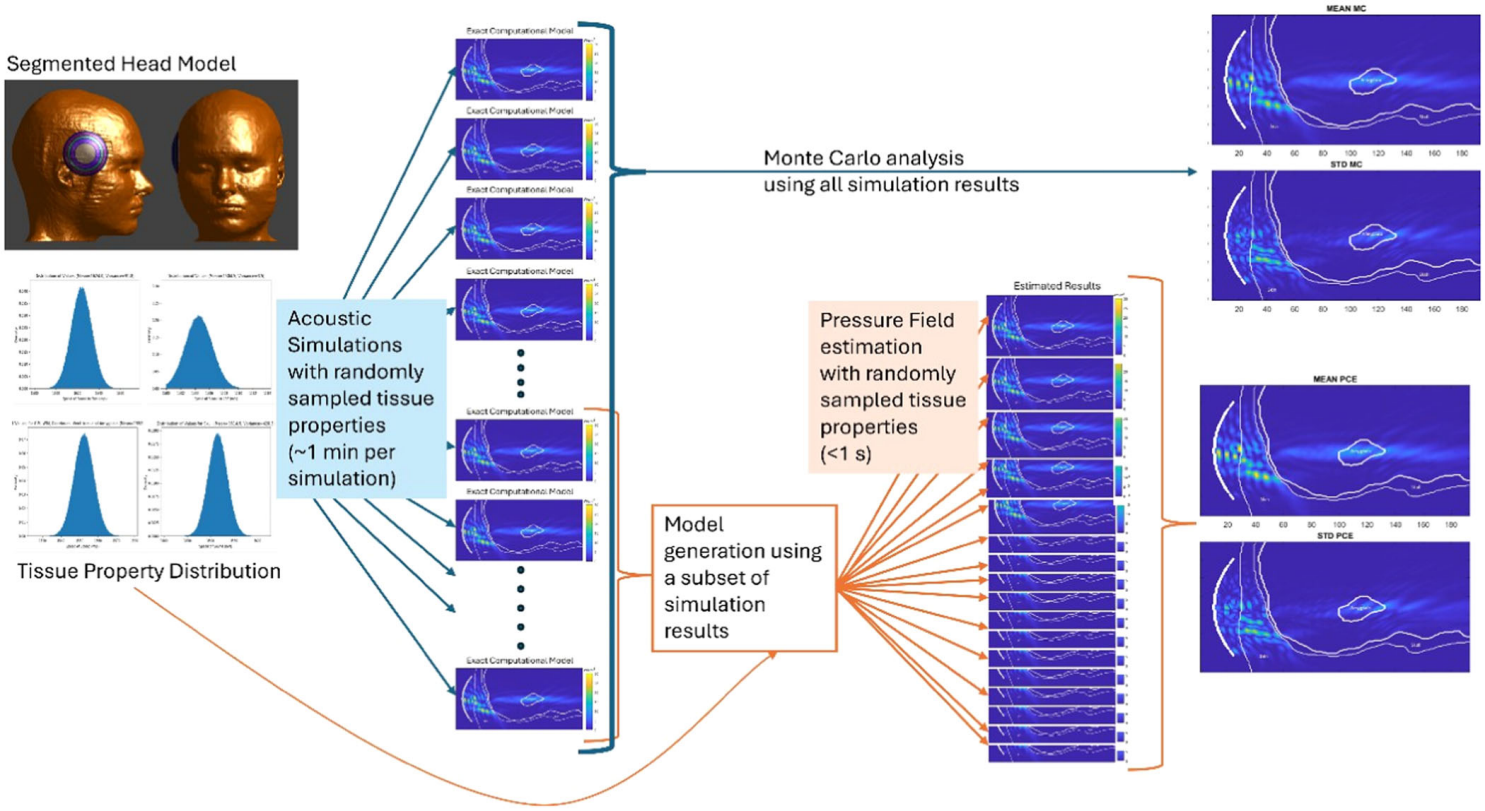
Schematic overview of the proposed framework for the rapid assessment of the impact of tissue property uncertainties.

A schematic overview of the proposed framework compared to the Monte Carlo method is shown in Figure 1. The proposed framework starts with the integration of the segmented head model with the FUS applicator model. To calculate the uncertainties arising from the tissue acoustic properties, a set of acoustic simulations with tissue acoustic properties randomly sampled from the expected ranges for each tissue type is performed. From the results of these simulations, we calculated the uncertainty in the acoustic intensity field using the Monte Carlo method. Using a set of simulations, we also generated a surrogate model using PCE, and the uncertainty in the acoustic intensity field was calculated. A more detailed explanation of the methods is given in the following sections.

### 2.1 Human Head Models

The human head models used in our study were obtained from the Population Head Model (PHM) Repository, which was developed at Iowa State University. The PHM Repository consists of 50 unique head models constructed from MRI images of healthy subjects between the ages of 22 and 35 distributed through the Human Connectome Project (Van Essen et al. 2012).

**Figure 2:**
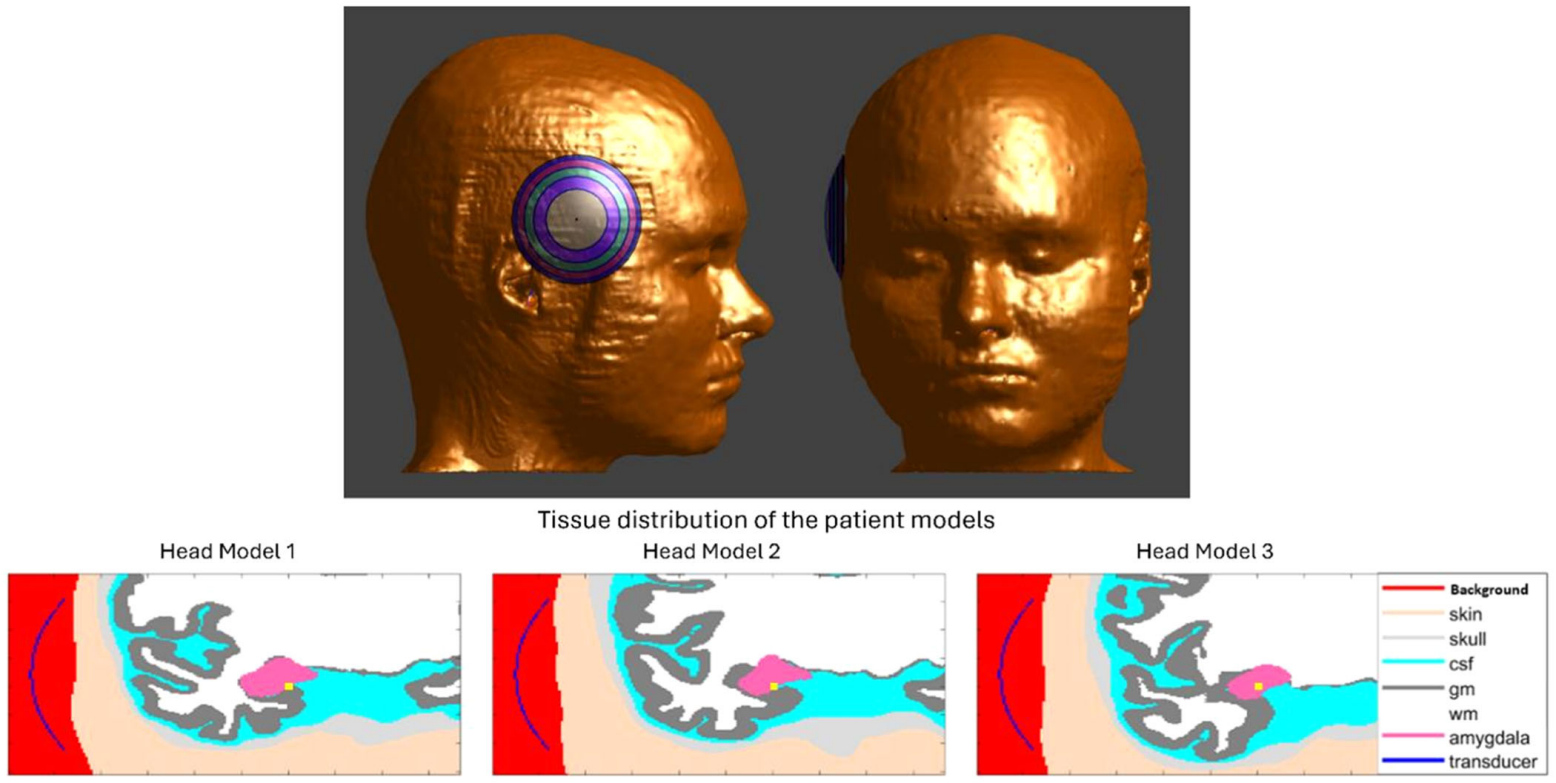
Representative image of the CTX‐250 transducer placed on the right side of the head (top row) and the tissue distribution of the three head models (bottom row) in the coronal slice passing through the transducer geometrical focus (shown with the yellow dot).

These head models were developed using the SimNIBS pipeline (Thielscher et al. 2015), to better understand the variation in subject response to stimulation from time‐varying magnetic fields in transcranial magnetic stimulation (TMS) studies (Lee et al. 2016, 2018). Each of these head models contain seven tissues: skin, skull, gray matter, white matter, cerebellum, cerebrospinal fluid, and ventricles. Since these head models lack a delineation of the amygdala, we have inserted the right amygdala lobe from the MIDA model (Iacono 2099) to the approximate location based on anatomical landmarks in these head models. Three head models used in our study are shown in Figure 2.

### 2.2 Ultrasound Applicator Model

The applicator model used in these simulations was based on NeuroFUS Cortical Focus Transducer (CTX)—250 (NeuroFUS, BrainBox Ltd, UK), which is a turnkey system that delivers low‐intensity (non‐thermal) focused ultrasound (FUS) for transcranial brain or transdermal nerve modulation. The CTX‐250 consists of four channels, has a 64 mm spherical radius, a 64 mm aperture diameter, and operates at center frequency of 250 kHz. The power levels delivered during the simulation study corresponded to a spatial peak pulse average intensity (*I*_sppa_) of 50 W/cm^2^, calibrated in free water. For each head model, the applicator is positioned on the right side of the head by touching the skin (see Figure 2), and it is aligned to have the geometrical focus in the center of the amygdala where possible, for example, physically, not clipping the tissue.

### 2.3 Acoustic Simulations

To calculate the pressure field generated by the CTX‐250 transducer operating at 250 kHz, acoustic simulations were performed in Sim4Life v7.2 (ZMT Zurich MedTech AG, Switzerland), which is a finite difference time‐domain solver of the 3D linear acoustic pressure wave equation (LAPWE; Kyriakou 2015), with the following formulation:

(1) $$\rho \nabla \frac{1}{\rho }\nabla p-\frac{1}{c^{2}}\frac{\partial^{2}p}{\partial t}-2\alpha \left(\sqrt{\frac{{4\alpha }^{2}}{\Omega^{2}}+\frac{1}{c^{2}}}\right)\frac{\partial p}{\partial t}=0$$

where *ρ* (kg/m^3^) is the density, *p* (Pa) is the acoustic pressure, *c* (m/s) is the speed of sound, *t* (s) is time, *α* (Np/m) is the absorption coefficient, and *Ω* (rad/s) is the angular frequency. A 0.5 mm cubic grid (one‐twelfth of a wavelength) was used in the whole computational domain (71 × 71 × 96.5, grid size 142 × 142 × 193) to ensure simulation accuracy. The tissue acoustic properties, attenuation, density, and speed of sound were assigned from the IT'IS database and given in Table 1 (Barth et al. 2007). The background medium was set to water. The model was simulated for 50 periods using multi‐CPU OpenMP processing on an i7‐12700H processor. Each simulation took approximately 55 s, excluding the voxeling process.

**Table 1:** Acoustic properties used in acoustic modeling.

Tissue	Density (kg/m^3^) [mean ± standard deviation]	Attenuation coefficient (Np/m)	Speed of sound (m/s) [mean ± standard deviation]
Amygdala	1045 ± 7	0.22	1552.5 ± 29.0
Cerebellum	1045 ± 7	0.42	1552.5 ± 29.0
CSF	1007 ± 1	0.025	1504.5 ± 3.5
Gray Matter	1044.5 ± 8	0.22	1552.5 ± 29.0
Skull	1908 ± 166	13.63	2813.7 ± 337.0
Skin	1109 ± 38	5.29	1624.0 ± 91.8
Ventricles	1007 ± 1	0.025	1504.5 ± 3.5
Water	1000	0	1500
White Matter	1041 ± 2	1.48	1552.5 ± 29.0

In each simulation, we perturbed the speed of sound and density for the different tissue types. The speed of sound and density perturbations was randomly drawn from normal distributions within the expected ranges for each tissue type, based on the literature (Barth et al. 2007).

### 2.4 Polynomial Chaos Expansion

We have implemented PCE using the Chaospy 4.3.13 library in Python 3.9.13 (Feinberg and Langtangen 2015). A polynomial basis was constructed that expresses the acoustic intensity field for a given tissue property uncertainties.

(2) $$Y=\sum_{n=0}^{M}c_{n}\Psi_{n}(\vec{\xi })$$

Here, *Y* represents the acoustic intensity field. *Cn* represents polynomial coefficients, *ψ*_*n*_ represents polynomial bases and $\vec{\xi }$ represents the parameter uncertainties. The total number of expansion terms (*M*) in equation (2) depends on the number of input parameters (*u*) and the degree of expansion, in other words, the order of the polynomial (*n*).

(3) $$M=\frac{\left(u+n\right)!}{u!n!}$$

In our model, we assumed that the tissue property uncertainties are independent, hence that the cross terms in the polynomials can be omitted. The total number of expansion terms is therefore given by

(4) M=u∗n+1

To find the coefficients of polynomial, we implemented the linear regression method using Scikit‐learn (Pedregosa et al. 2011). Linear regression is a “nonintrusive” PCE method, i.e., it does not require any modification to the underlying model. Instead, the model is treated as a black box and a PCE expansion is created using the simulations as model output.

**Figure 3:**
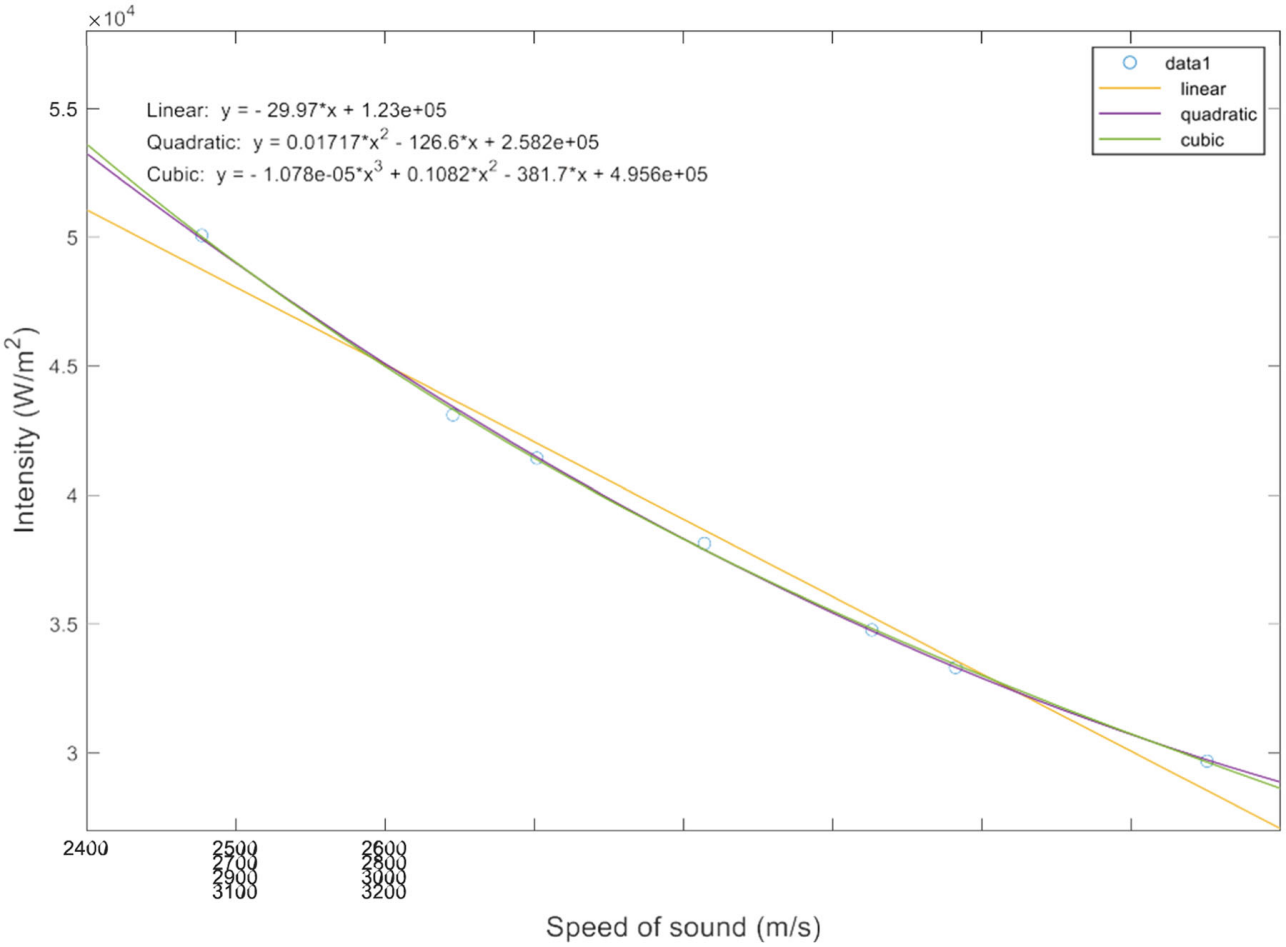
The variation of the acoustic intensity field values at the geometric focus as a function of the speed of sound of the skull.

Figure 3 shows the variation of the acoustic intensity field values at the geometric focus as a function of the speed of sound of the skull. The data shows a continuous and smooth trend which can be accurately represented using a second‐order polynomial fit. Therefore, a second‐order polynomial basis (*n* = 2) was selected to construct the surrogate model, in line with the results obtained for problems of similar complexity in the literature (Groen et al. 2023). Our head models consist of eight different tissues (*u* = 8), leading to 17 polynomial terms in total for the case when only the speed of sound uncertainty is considered. To test the effect of sample size, we subsequently used 5, 10, 17, 20, 30, and 40 simulation samples for creating the surrogate models. The number of samples less than 17 represents an under‐defined problem and the number of samples more than 17 represents an over‐defined problem. To show that our proposed method can capture the propagation of uncertainty associated with other types of acoustic properties, we extended our analysis by modelling density uncertainties in addition to the speed of sound uncertainties. With 16 uncertainties, resulting in a total of 33 polynomial terms. We ran the PCE model with 40 and 50 samples.

### 2.5 The Effect of Tissue Uncertainty Evaluation and Monte Carlo Simulations

Two hundred and fifty Monte Carlo simulations were used to investigate the effects of tissue acoustic property uncertainties on our model predictions. These properties were randomly sampled from their respective distributions and mean and standard deviation in each voxel were calculated. For comparison, we also used a surrogate model to generate 250 predictions of the model output, using the same random inputs used for the Monte Carlo simulations and we calculated the mean and standard deviation of the model output in each voxel.

### 2.6 Accuracy Evaluation

In LIFUS, large parts of the calculation domain remain unaffected by the acoustic intensity field distribution generated by the transducer. Hence, we limited the acoustic intensity field accuracy analysis to those voxels where the intensity exceeded 1 W/cm^2^, corresponding to 2% of the maximum spatial peak pulse average intensity value (50 W/cm^2^) in free water.

Within these specified voxels, we established a set of success criteria, defined as achieving a minimal difference of less than 3% between computed and estimated values, constituting a pass/fail metric. To determine the success rate, 50 individual simulations with random speed of sound samples were performed, and the acoustic intensity field was estimated using the surrogate model with the same input values. We computed the success rate for each simulation and subsequently calculated the overall success rate across all 50 simulations. To quantitatively compare uncertainty prediction accuracy between Monte Carlo and the surrogate model, we used the root mean squared percentage error (RMSPE).

(5) $$\mathrm{RMSPE}=\sqrt{\frac{1}{n}\sum_{i=1}^{n}{\left(\frac{y_{i}-{\hat{y}}_{i}}{y_{i}}\right)}^{2}\times 100}$$

where *yi* is the computed uncertainty value by Monte Carlo method and *ŷi* is the estimated uncertainty value by PCE.

## 3 Results

**Figure 4:**
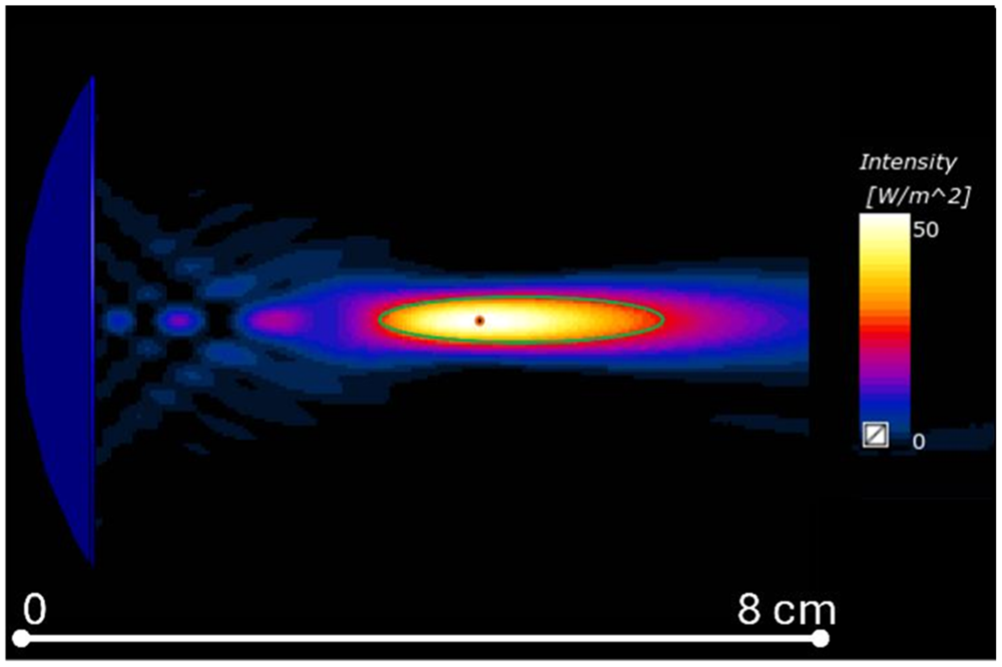
Cross‐section of the acoustic intensity field in free‐water and the focus size and shape (outlined with a green ellipse, 38 mm in propagation direction, 6 mm in the transverse directions).

The acoustic intensity field generated in water by the CTX‐250 transducer is shown in Figure 4. The focus peak was located at 64.4 mm from the transducer center and the input power was adjusted to achieve 50 W/cm^2^ at the focus point. The focus had an elliptical shape. It extended 38 mm in propagation direction, and 6 mm in both transverse directions.

**Figure 5:**
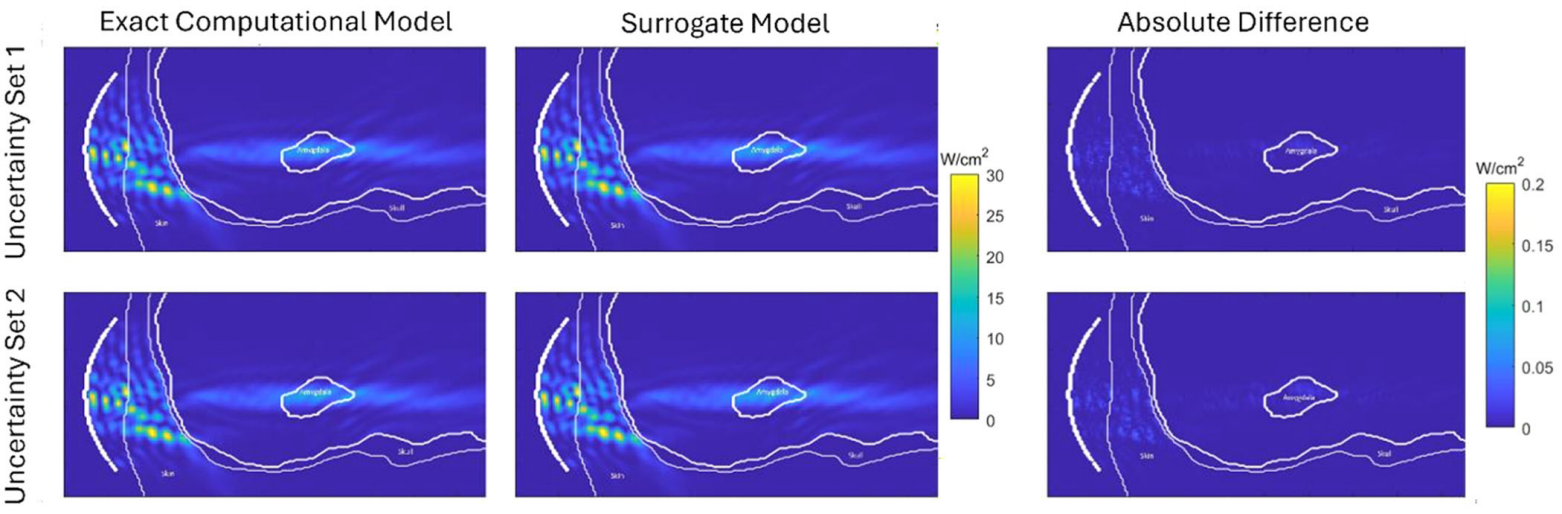
Example acoustic intensity field distribution in the coronal slice passing through the transducer focus calculated by the exact computational model (first column), prediction by the surrogate model (second column), and the difference (third column) for two different uncertainty sets (top and bottom row). The isolines show the outlines of the amygdala, skull, skin and the transducer (on the left).

In Figure 5, an example of acoustic intensity field distributions estimated by the surrogate model based on the acoustic wave simulations is visualized for head Model 1 using two different uncertainty sets. Table 2 shows the success rate results for three different head models. As expected, the success rate of all three models increases with the number of training points. For all three head models, the surrogate model achieves high success rates (> 90%) after 20 training points for 8 uncertainties. The generation of the surrogate model using 20 training points for the entire simulation domain (~4 M voxels) took around 1 h in total (18 min for running 20 acoustic simulations and 45 min to calculate the polynomial coefficients) using a laptop with an i7‐12700H processor.

**Table 2:** Model success rate as a function of training points.

Number of uncertainties	Number of training points	Success rate (%) for Head Model 1	Success rate (%) for Head Model 2	Success rate (%) for Head Model 3
8	5	58.1	49.2	45.2
8	10	70.7	60.5	72.3
8	17	79.0	75.9	85.2
8	20	91.0	90.3	96.1
8	30	97.0	94.9	96.2
8	40	97.6	98.3	96.9
16	40	82.0	83.3	86.9
16	50	95.0	94.1	94.8

**Figure 6:**
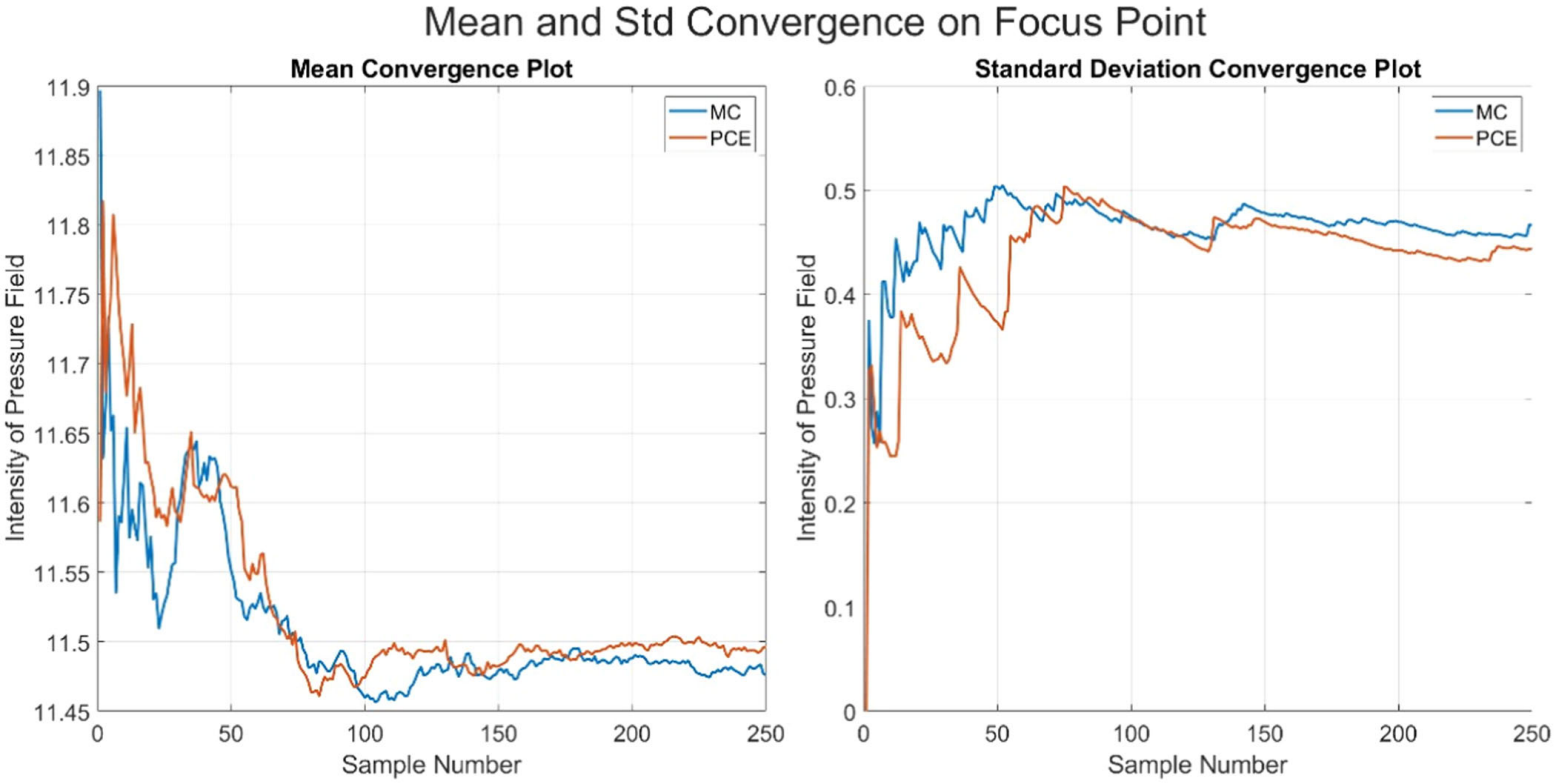
Convergence plot on the focus point for Monte Carlo and PCE Monte Carlo: (left) convergence plot of the mean value, (right) convergence plot of the standard deviation.

Figure 6 shows the convergence of the estimated uncertainty at the focus point of the ultrasound transducer for the Monte Carlo simulations and the surrogate models generated using 20 samples. The estimated uncertainty converges around 100 random samples.

**Figure 7:**
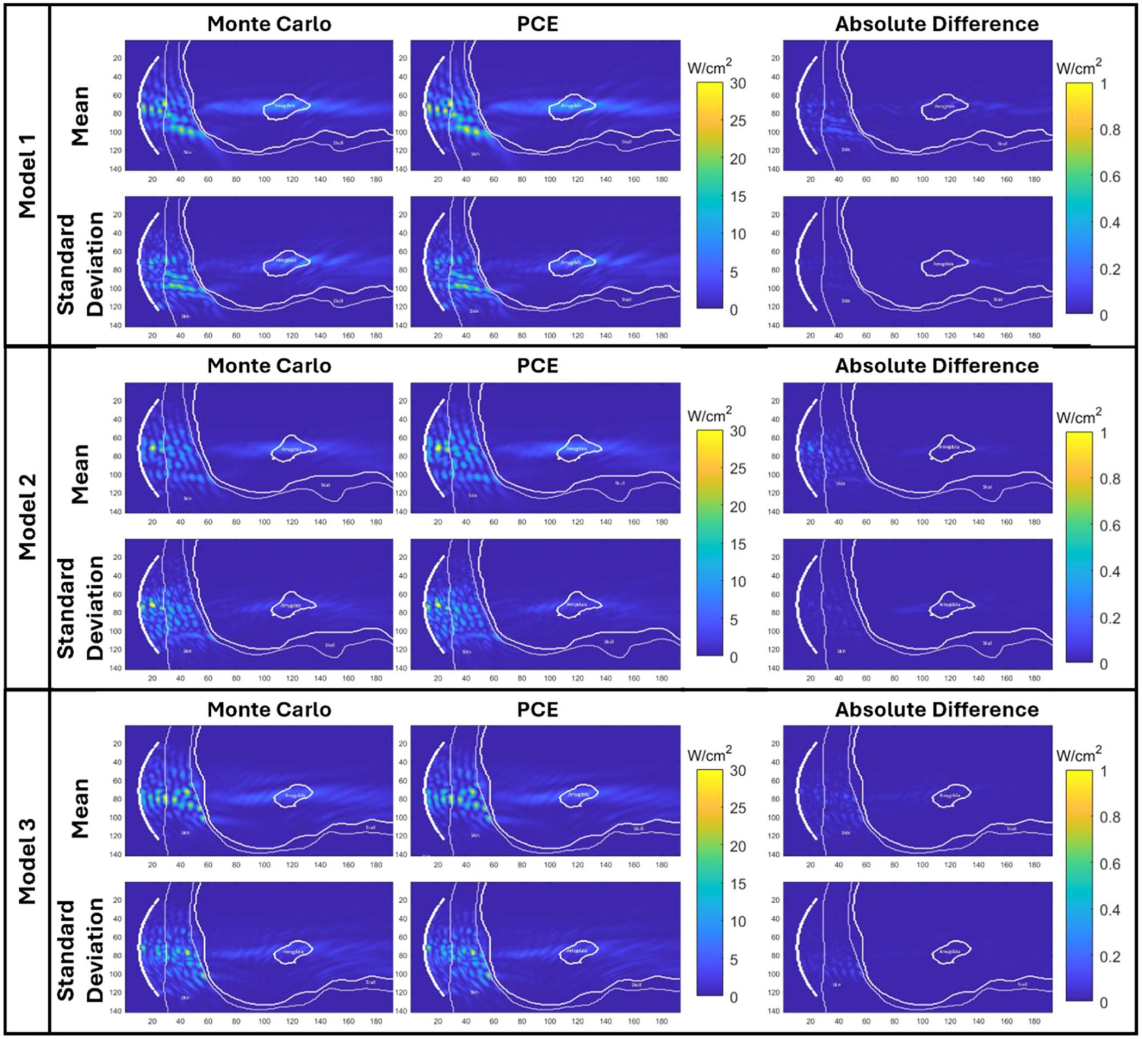
Mean and standard deviation per head model for the acoustic intensity field in the coronal slice passing through the transducer focus calculated by the Monte Carlo computational model (first column), PCE (second column), and the difference (third column) for mean (first row) and for standard deviation (second row).

In Figure 7, example slices for mean and standard deviation for the acoustic intensity field calculated by the Monte Carlo simulations and the surrogate modeling and the absolute difference between them has been illustrated for all models. We have found 1%, 1.2% and 1.1% RMSPE in the mean acoustic intensity field for Head Models 1, 2, and 3, respectively. The maximum error in the mean was 0.18 W/cm^2^ for Head Model 1, 0.47 W/cm^2^ for Head Model 2, 0.43 W/cm^2^ for Head Model 3. We have found 3.6%, 8.3% and 8.6% RMSPE in the standard deviation for Head Model 1, 2, and 3, respectively. The maximum error in the standard deviation was 0.09 W/cm^2^ for Head Model 1, 0.20 W/cm^2^ for Head Model 2, 0.28 W/cm^2^ for Head Model 3.

## 4 Discussion

In this study, we developed and evaluated a surrogate model based on PCE to efficiently quantify the uncertainty in the LIFUS acoustic intensity field caused by multiple tissue acoustic property uncertainties. We compared our method against the traditional Monte Carlo method using three different head models. We also investigated the effect of the number of samples on the accuracy of the surrogate model. Our results showed that the PCE surrogate model could accurately estimate the mean and standard deviation of the acoustic intensity field with high accuracy. The RMSPE in the mean acoustic intensity field was less than 1.5% for all three head models, and the maximum error in the mean was less than 0.5 W/cm^2^ (< 1% of the focus peak intensity in water). The RMSPE in the standard deviation was less than 9% for all three head models, and the maximum error in the standard deviation was less than 0.3 W/cm^2^.

We have found that the accuracy of the surrogate model significantly improves with an increasing number of training samples. For the specific case that we have investigated with eight input uncertainties, the under‐defined case is represented by number of samples less than 17, and over‐defined case is represented by number of samples more than 17. With a slight over‐defined sampling scheme using 20 samples, our method was able to create a surrogate model with high accuracy (> 90%). A further increase of the input sample size to 30 is sufficient to improve the surrogate model accuracy to over 95%. This accuracy can be further improved since we have chosen our input samples randomly. A pseudo‐random sample set limited by the mean and standard deviation limits of the input samples can be used to guide the sample selection set.

The main advantage of the PCE method over the Monte Carlo method is computational efficiency. In our case study, compared to the Monte Carlo method, which requires 100 samples and takes about 90 min, the PCE method can produce the same results in 60 min for the entire simulation domain. A further increase in speed can be achieved by restricting the generation of the surrogate model to the voxels of interest, since the large part of the simulation domain, although required for the acoustic simulations, is not of interest for uncertainty assessment. Our calculation domain in this study consisted of 4 M cells, while the amount of voxels that passes a sound intensity threshold of 1 W/cm^2^, were 235k for Head Model 1, 207k for Head Model 2, and 248k for Head Model 3. Restricting the surrogate model calculation to the voxels with a specified intensity will reduce the time to calculate the polynomial coefficients from 45 min to ~2.5 min, resulting in 17.5 min to calculate the uncertainties. Hence, 80% gain in time is possible using this framework. Note that Monte Carlo simulations always require simulation of the whole domain for every simulation. It is also important to note that not all parameters have the same effect on the simulation results.

For example, skull uncertainty has a dominant effect on acoustic simulations, while tissues with less uncertainty, such as CSF, have a minimal effect. This should be carefully considered when constructing polynomials for each voxel. Selecting the polynomial order based on the influence of uncertainty on a given voxel can improve accuracy and reduce the time required to calculate the polynomial coefficients. Furthermore, the real power of the PCE method comes into play when individual uncertainties are to be investigated. Once the surrogate model has been generated, the number or range of input samples can be varied at will. On the other hand, the Monte Carlo method requires all the simulations needed to analyze the new uncertainty to be rerun.

Another advantage of the pipeline we generated for uncertainty assessment is that the PCE method can handle multiple uncertainties and treat the acoustic simulations as a black box. Recent studies have investigated efficient calculation of the effect of skull uncertainties in transcranial ultrasound simulation (Stanziola et al. 2023). Here, we show the scalability of our method by assessing multiple uncertainties and acquiring highly accurate results. Since our method treats the acoustic simulations as a black box, we can easily scale our method for thermal safety assessment, as well as include thermal property uncertainties. Hence, PCE‐based uncertainty assessment is a promising tool to be integrated with routine LIFUS treatments.

## 5 Conclusion

In conclusion, we have developed and evaluated a novel PCE surrogate model to quantify the uncertainty in the acoustic intensity field caused by tissue acoustic property uncertainties in LIFUS applications for neuromodulation. Our results demonstrate that the PCE surrogate model can accurately estimate the mean and standard deviation of the acoustic intensity field with high accuracy and computational efficiency, outperforming the traditional Monte Carlo method. The accuracy and efficiency of the PCE surrogate model make it a promising tool for LIFUS applications. Furthermore, the flexibility of our tool enables the extension of its application to other safety assessments, such as thermal, in LIFUS applications.

## Conflicts of Interest

The authors declare no conflicts of interest.
